# (1, 3)-beta-D-Glucan in bronchoalveolar lavage fluid: a useful biomarker in diagnosis of invasive pulmonary infection caused by *Hormographiella aspergillata*?

**DOI:** 10.1186/s13000-024-01589-9

**Published:** 2024-12-28

**Authors:** Haiyan Ye, Jinhui He, Jing Huang, Patrick Chu, Junru Liu, Rosana Wing-Shan Poon, Fanfan Xing, Simon Kam-Fai Lo, Ricky Wing-Tong Lau, Jasper Fuk-Woo Chan, Susanna Kar-Pui Lau, Kelvin Hei-Yeung Chiu

**Affiliations:** 1 Department of Infectious Disease and Microbiology, The University of Hong Kong-Shenzhen Hospital, Shenzhen, Guangdong China; 2 Department of Hematology, The University of Hong Kong-Shenzhen Hospital, Shenzhen, Guangdong China; 3 Department of Pathology, The University of Hong Kong-Shenzhen Hospital, Shenzhen, Guangdong China; 4 Department of Microbiology, Queen Mary Hospital, Pokfulam, Hong Kong Special Administrative Region China; 5 State Key Laboratory of Emerging Infectious Diseases, Department of Microbiology, School of Clinical Medicine, Li Ka Shing Faculty of Medicine, Carol Yu Centre for Infection, The University of Hong Kong, Pokfulam, Hong Kong Special Administrative Region China

**Keywords:** *Hormographiella aspergillata*, (1,3)-beta-D-Glucan, Invasive fungal infection, Amphotericin B

## Abstract

*Hormographiella aspergillata* is a rare hyaline mold causing invasive fungal infection in humans, until the frequent use of antifungal prophylaxis in immunocompromised hosts. Due to the high mortality of *H. aspergillata* infection, early recognition and treatment are crucial. Previous case reports suggested that serum (1,3)-beta-D-Glucan (BG) is one of the diagnostic aids for *H. aspergillata* infection. Here we report for the first time a case of pulmonary *H. aspergillata* infection with a negative serum BG but positive bronchoalveolar lavage fluid (BAL) BG. This may suggest that BAL BG is a useful and additional microbiological marker for prompt identification of this fatal invasive fungal infection (IFI). But it should be interpreted together with the clinical presentation, imaging, and other laboratory results.

## Introduction

Invasive fungal infection (IFI) is a serious complication in patients with hematological malignancies. With the use of antifungal prophylaxis in immunocompromised hosts, there is a change in the etiology of fungal infection from *Aspergillus* and *Candida* to more resistant fungi such as *Mucorales*,* Fusarium*,* Scedosporium*, and *Basidiomycetes* [1, 2]. Pulmonary infection due to *Hormographiella aspergillata* is rare but associated with a high mortality rate, leading to delays in diagnosis and treatment [3]. The diagnosis of invasive fungal infection is often difficult, because of risks associated with invasive procedures such as lung biopsy or bronchoalveolar lavage in patients with coagulopathies, low sensitivity of routine fungal culture, together with the long turn-around time in confirmation of fungal identity due to lack of expertise and the need of further tests for confirmation. Previous case reports have reported that serum BG levels were elevated in patients with *H. aspergillata* infection, suggesting it is an important marker of *H. aspergillata* infection [4–7]. Here we report the first case of pulmonary *H. aspergillata* infection with a negative serum but a positive BAL BG, suggesting that BAL BG may be a useful and additional tool for prompt diagnosis of this fatal IFI.

## Presentations

A 34-year-old Chinese female, with newly diagnosed acute lymphoblastic leukemia, was admitted to our hospital for chemotherapy . Prior to admission, she experienced a dry cough and persistent neutropenic fever for 15 days, without improvement despite broad-spectrum antibiotics coverage with biapenem, amikacin, and itraconazole in another hospital. Chemotherapy was administered according to the GRAALL-2003 regimen [8] (Intravenous vincristine, daunorubicin, pegaspargase, and cyclophosphamide; oral prednisone; intrathecal methotrexate, cytarabine, and dexamethasone) from day 6 to day 34 of hospitalization. She enjoyed good past health with no known drug allergies. She had no personal history or contact history of pulmonary tuberculosis. She kept no pets at home and had never used any over-the-counter medication or traditional Chinese medicine. She was a non-smoker and a social drinker. Her body temperature was 38.7 °C on admission, with a heart rate of 110 beats per minute, blood pressure of 112/69 mm Hg, respiratory rate of 28 breaths per minute, and oxygen saturation of 80% in room air. There were no palpable lymphadenopathies or finger clubbing. Abdominal, respiratory, and cardiovascular examinations were all unremarkable.

**Fig. 1:**
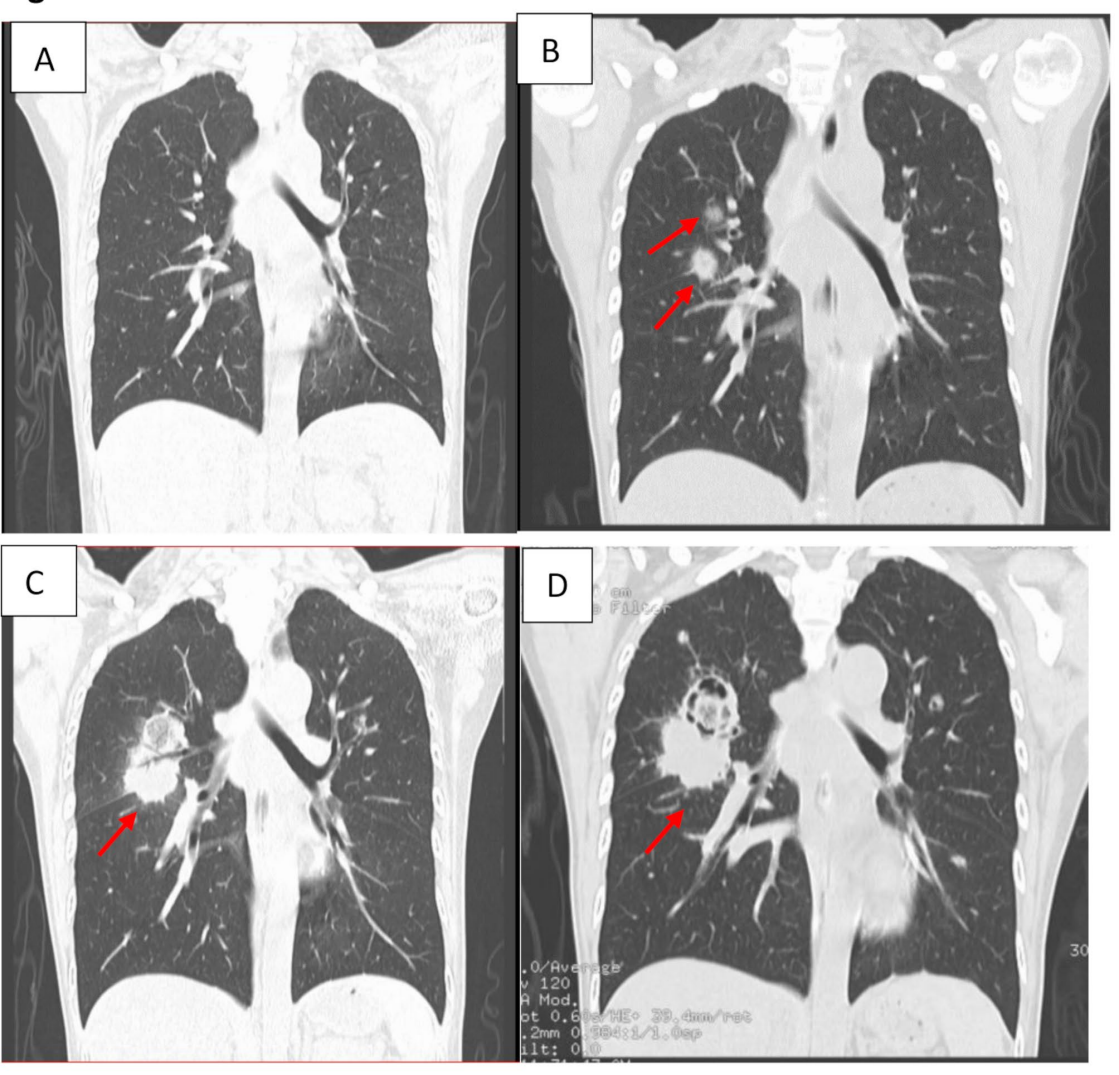
CT thorax (sagittal view) showing the progression of the lung lesions (arrows). **A**: No signs of pneumonia and pulmonary lesions on day 9; **B**: Two new pulmonary nodules (the biggest one is 1.9 × 1.5 cm) located in the right middle lobe surrounded by a halo of ground-glass opacity on day 21; **C**: Progression of the right middle lung mass (the biggest one is 3.5 × 5.2 cm) with cavitation on day 33; **D**: Progression of the right middle lung mass(3.6 × 5.2 cm) with cavitation on day 41

On day 9 of admission, she remained neutropenic and febrile despite broad-spectrum antibiotics (imipenem, vancomycin, trimethoprim-sulfamethoxazole, doxycycline, and levofloxacin) and itraconazole prophylaxis. Computed tomography (CT) of the thorax showed no signs of pneumonia and pulmonary lesions (Fig. 1A). In view of persistent fever, itraconazole was switched to micafungin for antifungal prophylaxis. However, fever still persisted despite adjustment of antifungal, with a repeated CT thorax (day 21) demonstrating two new pulmonary nodules in the right middle lobe with halo signs (Fig. 1B). Blood culture, sputum for acid-fast bacilli (AFB) smear, and sputum for bacterial culture were unremarkable. Sputum fungal culture was not ordered and performed. *Mycobacterium tuberculosis* DNA was not detected in the sputum by PCR. Serum galactomannan and BG were negative on Day 22. Bronchoscopy with biopsy was not initially performed due to refusal by the patient and her family, therefore micafungin was empirically switched to caspofungin, with the subsequent addition of voriconazole. However, reassessment CT thorax (day 33) still showed a progression of the right middle lung mass with cavitation (Fig. 1C). A bronchoscopy with lung biopsy was subsequently performed on the following day (day 34), and a mucous plug containing purulent secretions in the right posterior segmental bronchus was removed and sent for microbiological investigations. Both BAL and lung tissue bacterial cultures were negative, with *Mycobacterium tuberculosis* DNA not detected in the specimens. BAL galactomannan antigen was negative, but BAL BG was positive (> 600 pg/mL). Antifungal was subsequently switched to amphotericin B deoxycholate, escalating from 5 mg daily to a treatment dose of 40 mg (1 mg/kg) daily (from day 34 to day 41) to reduce the chance of acute hypersensitivity reaction. Despite the recovery of her neutrophil level and the administration of amphotericin B deoxycholate, the lung lesions continued to progress (Fig. 1D) with her clinical condition deteriorating rapidly, and the patient died from severe hemoptysis and respiratory failure on day 44.

## Mycology and histology

**Fig. 2:**
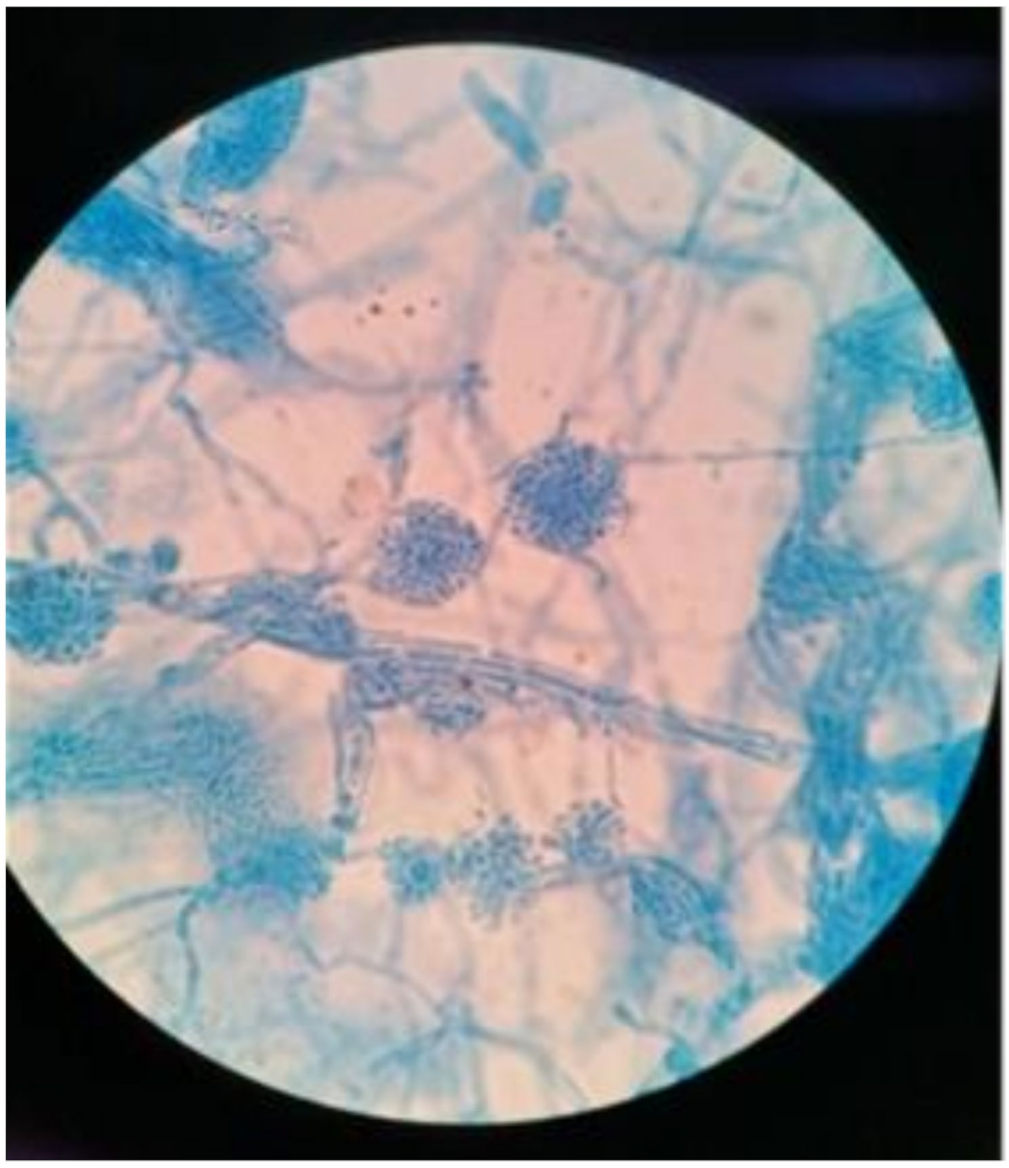
Staining with lactophenol blue demonstrating septate conidiophores with clusters of smooth-walled, hyaline, and cylindrical arthroconidia (10 × 100)

**Fig. 3:**
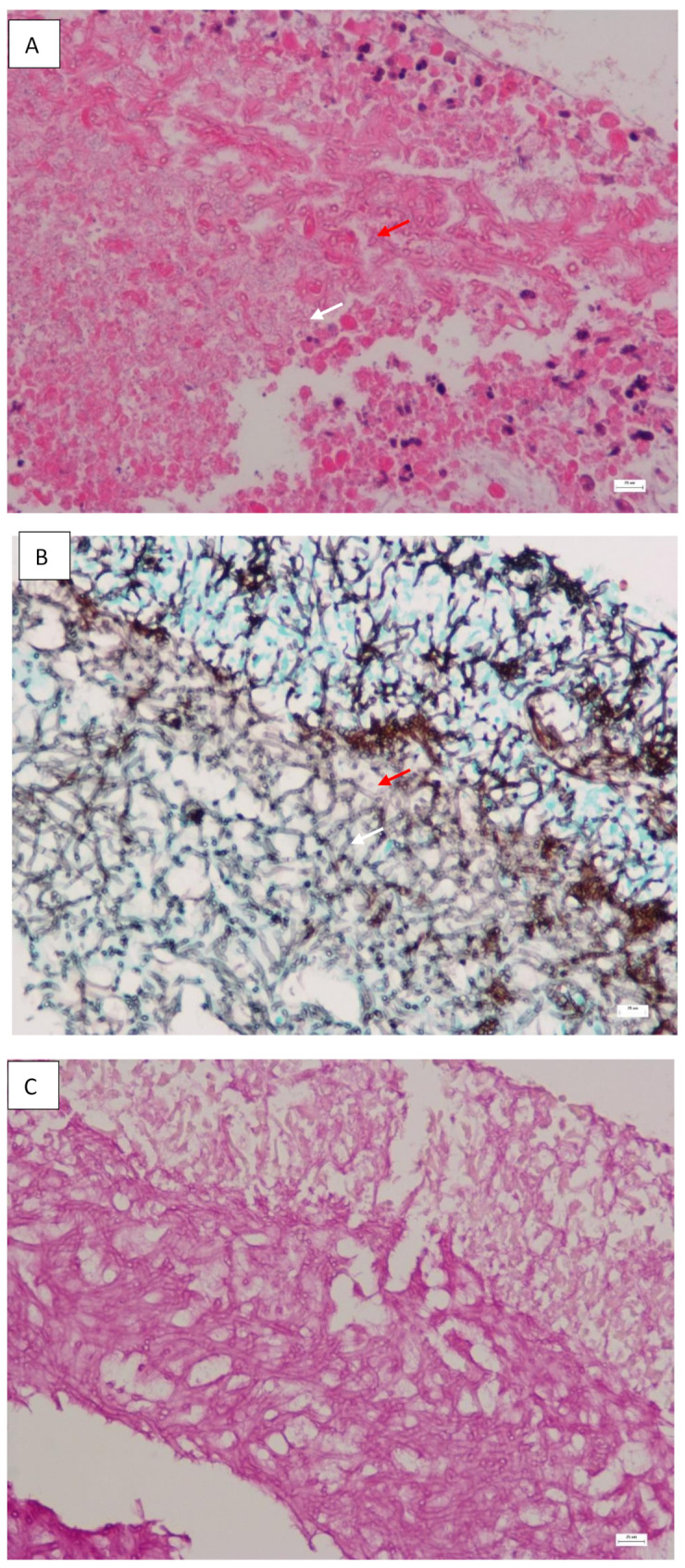
Histology of lung biopsy showing septate (red arrow) and branched hyphae with the formation of clews (white arrow); **A**: Hematoxylin and Eosin stain (magnification,× 400); **B**: Gomori-Grocott stain (magnification × 400); **C**: Periodic Acid-Schiff stain (magnification × 400)

After incubating the BAL and lung tissue on Sabouraud dextrose agar at 37℃ for 48 h, white to cream-colored colonies and cottony aerial mycelium were observed. All inoculated media (Sabouraud dextrose agar, chocolate agar, and blood agar) yielded pure cultures of this fungus in the following days. Staining with lactophenol blue demonstrated septate conidiophores with clusters of smooth-walled, hyaline, and cylindrical arthroconidia (Fig. 2). Pan-fungal PCR and subsequent sequencing using the internal transcribed spacer (ITS) region of the rRNA gene of the isolates were performed. Based on the Basic Local Alignment Search Tool (BLAST) search of the sequence on the ITS region of the isolated fungus, the homology of *Coprinopsis cinerea* culture CBS 338.69 strain (GeneBank Accession No: MH878445.1) was 100.0% (365/ 365 bp), therefore the organism was identified as *Coprinopsis cinerea* (the teleomorph of *Hormographiella aspergillata*). Histology of the lung tissue revealed hyaline, septate, and branched hyphae with evidence of angioinvasion (Fig. 3). Antifungal susceptibility testing was performed by the E-test method on Sabouraud dextrose agar, with the minimal inhibitory concentrations (MIC) of voriconazole and amphotericin B being 32 μg/mL and 0.94 μg/mL, respectively.

## Discussion

*H. aspergillata* belongs to the Basidiomycota division of fungi, and is the asexual form of *Corprinopsis cinerea*, an edible mushroom usually found in compost and other nutrient-rich substrates. *H. aspergillata* is a rare hyaline mold causing invasive fungal infection in humans. Twenty-seven cases were reported in the literature from 23 publications, with infection occurring mainly in patients with hematological malignancies [3–7, 9–26]. The majority of the patients were diagnosed in Europe, with other cases documented in the United States, Japan, and India. Most cases of *H. aspergillata* infection have been reported as pulmonary and disseminated infections, but the involvement of the skin, central nervous system, eye, and spleen was also reported in immunocompromised patients [5, 6, 12, 15]. Endocarditis and endophthalmitis have been described in immunocompetent patients [10, 20, 25, 26]. The mortality rate of *H. aspergillata* infection was high (78%), likely related to the delay in diagnosis and initiation of effective antifungal agents [3]. Patients who survived were managed with prompt invasive diagnostic procedures such as bronchoscopy and lung biopsy for respiratory infection, adequate surgical debridement of involved areas, prompt initiation of broad-spectrum antifungal therapy, as well as recovery of neutrophils in patients with underlying hematological malignancies.

Most cases of *H. aspergillata* infection were diagnosed by histology with confirmation by molecular techniques such as PCR and sequencing. Due to the high mortality of *H. aspergillata* infection, early recognition and treatment are crucial. We attempted to evaluate the value of galactomannan, BG, and glucuronoxylomannan (GXM) antigens from previous studies to facilitate the early diagnosis of *H. aspergillata* infection. GXM is the capsular antigen of *Cryptococcus neoformans*, that is widely used for the diagnosis of cryptococcosis. Cross-reactions with GXM have been described in the members of *Basidiomycetes* such as *Trichosporon* and *Coprinopsis cinerea* [27]. However, another study has demonstrated that the culture supernatants of *H. aspergillata* produce galactomannan and BG but not GXM [4]. This concurs with the two reported cases of *H. aspergillata* infection that the serum GXM antigens were negative [19, 20]. Despite the in vitro result of positive galactomannan, the diagnostic utility of galactomannan is doubtful, as all documented cases of *H. aspergillata* infection have negative galactomannan assay. The situation of the use of BG for diagnosis is different, with an earlier study showing that BG is an important component of the cell wall of *Coprinus cinereus* [28]. In five reported cases of *H. aspergillata* infection with serum BG checked, four (80%) of them had positive serum BG (greater than 500pg/mL) [3–7]. Serum BG was also found to be positive seven days prior to the development of radiological changes and at least one month prior to the identification of *H. aspergillata* in one of the case reports [7]. In our case, serum BG was negative despite positive radiological findings, but the BAL BG was found to be strongly positive (> 600 pg/mL). Although *Candida spp.* colonization or overgrowth at the lower respiratory tract can lead to false positive BAL BG results, yeasts have never been cultured from the respiratory specimens in our case, which further supports *H. aspergillata* as the cause of positive BAL BG in this case. Although BAL BG has a similar sensitivity to BAL galactomannan in the diagnosis of invasive aspergillosis and fungal infection (71%), it exhibits inferior specificity [29]. A positive BAL BG should be interpreted together with the clinical presentation, imaging, and other laboratory results for the diagnosis of IFI [30].

Currently, there is no standardized antifungal susceptibility protocol, clinical breakpoints, and treatment guidelines for *H. aspergillata*. Based on previous in vitro data, Jonathan T et al. summarized the antifungal susceptibility profile of 16 *H. aspergillata* clinical isolates reported in the literature, most cases exhibited a relatively low MIC range of voriconazole (0.015 to 1 mg/L) and amphotericin B (0.03 to 2 mg/L), except one case of amphotericin B MIC was 32 mg/L, while the MIC range of caspofungin (0.5 to 32 mg/L) and micafungin (0.25 to 16 mg/L) were relatively higher, indicating that voriconazole and amphotericin B are important antifungal agents for the treatment of *H. aspergillata* infection [3]. Although the method of susceptibility testing used was not standardized, the voriconazole MIC of our *H. aspergillata* isolate was higher than the usual reported range in the literature, which concurs with the fact that our patient did not respond to voriconazole treatment. Breakthrough infection after empirical voriconazole treatment has been reported on three occasions for *H. aspergillata* infection [11, 14, 17]. Based on the above factors and experience, voriconazole may not be a reliable primary regimen for *H. aspergillata* infection. Amphotericin B should be considered as the first-line antifungal agent.

Voriconazole or liposomal amphotericin B are preferred antifungal agents for the treatment of IFI. Amphotericin B is a polyene antifungal agent with activity in vitro against a wide variety of fungal pathogens [11, 14, 17], including most *Candida* spp, most hyaline and dematiaceous molds, and all dimorphic fungi. Liposomal amphotericin B has been introduced in the market to reduce the toxicities associated with amphotericin B deoxycholate. However, liposomal amphotericin B is not affordable for many patients in developing countries. According to FDA’s labeling resources for human prescription drugs, administration of amphotericin B deoxycholate should start with a test dose of 1 mg intravenously over 20 to 30 min, and healthcare workers should observe for any acute hypersensitivity reactions within 30 min. If no reactions were observed, then the remaining treatment dose can be administered. However, the practice in China is different. Amphotericin B deoxycholate is usually administered at a dosage of 1–5 mg for the first day, with a stepwise increase by 5 mg daily or alternate day based on the patient’s tolerance, until the treatment daily dosage of 0.6-1 mg/kg is reached. Further studies are required to compare the tolerance of patients with the two-dosing approach using the amphotericin B deoxycholate manufactured in China. The stepwise dosing approach delays the achievement of therapeutic concentration, leading to a delay in the management of invasive *H. aspergillata* infection in our patient. Therefore, we believe that in the treatment of severe and life-threatening fungal infection in neutropenic patients, the FDA recommendations of amphotericin B deoxycholate administration should be followed to ensure the timely administration of therapeutic concentrations of antifungal agents.

## Conclusion

BAL BG is a useful microbiological investigation for the diagnosis of invasive fungal infection as demonstrated in our case of fatal pulmonary *H. aspergillata* infection, but it should be interpreted with an assessment with clinical presentation, imaging, and other laboratory results. Amphotericin B, instead of voriconazole, should be recommended as the first-line treatment for *H. aspergillata* infection. In the treatment of severe and life-threatening fungal infection in neutropenic patients, the FDA recommendations (test dose then treatment dose) should be followed to ensure the timely administration of therapeutic concentrations of antifungal agents.

## Data Availability

No datasets were generated or analysed during the current study.

## Abbreviations

BAL: Bronchoalveolar lavage fluid
BG: (1,3)-beta-D-Glucan
CT: Computed tomography
GXM: Glucuronoxylomannan
*H. aspergillata*: *Hormographiella aspergillata*
IFI: Invasive fungal infection
ITS: Internal transcribed spacer
